# Association between sociodemographic variables and delayed patient presentation among surgical neuro-oncology patients in Mexico City: a single institution experience

**DOI:** 10.1007/s11060-024-04827-8

**Published:** 2024-09-24

**Authors:** Maria A. Punchak, Jose Alfonso Alvarez-Castro, Jonathan Ramos Escalante, Keren Magaly Aguilar Hidalgo, Mauricio Macias Zamarripa, Xymena Dominguez Navarrete, Fernando Castro Soto, Mackenzie Castellanos, Sergio Moreno-Jiménez, Michael T. Lawton, Alfredo Quinones-Hinojosa, Sonia Iliana Mejía Pérez

**Affiliations:** 1 Department of Neurosurgery, University of Pennsylvania Health System, Philadelphia, PA 19146 USA; 2 Department of Neurosurgery, Instituto Nacional de Neurología y Neurocirugía, Mexico City, Mexico; 3 Perelman School of Medicine, University of Pennsylvania, Philadelphia, PA USA; 4 Department of Neurosurgery, Barrow Global, Barrow Neurological Institute, Phoenix, AZ USA; 5 Department of Neurosurgery, Mayo Clinic, Jacksonville, FL USA

**Keywords:** Global neurosurgery, Middle-income countries, Neuro-oncologic care, Surgical capacity, Treatment delays

## Abstract

**Purpose:**

Mexico has the second highest incidence of central and peripheral nervous system cancer cases in Latin America, but clinical and research resources to improve oncologic care are biased towards high-income countries. We carried out a retrospective study to identify sociodemographic factors associated with more severe clinical presentation among surgical neuro-oncology who underwent surgery at a major public referral hospital in Mexico City.

**Methods:**

The hospital electronic medical record was reviewed to identify all surgical neuro-oncology patients who underwent surgery between January 1 and December 31, 2022. Descriptive statistics were used to characterize the patient population and outcomes; statistical analysis was performed to determine association between sociodemographic variables and advanced clinical presentation.

**Results:**

A total of 366 neuro-oncology patients underwent surgery during the study period. The median patient age was 48 (IQR 17–83). The majority of patients were female (60.1, *n* = 220), single (51.4%, *n* = 188), and 29.2% (*n* = 107) endorsed being the primary provider for their family. The median number of dependents per patient was 4 (IQR 2–50), while the median monthly income was 10269 Mexican pesos (MXN) (IQR 2000–13500] and the median travel distance to INNN was 49 km (IQR 22–174). On multivariate analyses, having a higher number of dependents was associated with increased odds of presenting with longer symptom duration (*p* = 0.01). Divorced/separated status was associated with increased odds of presenting with tumors > 35mL in volume (*p* = 0.04). Primary provider (*p* = 0.01) and higher average monthly income (*p* = 0.03) was associated with decreased odds of presenting with tumors > 35mL.

**Conclusions:**

This is the first study to recognize that certain sociodemographic factors are associated with more severe clinical presentation among surgical neuro-oncology patients. Further studies are needed in order to decern specific causes for delayed presentation in this patient population in order to create targeted interventions and decrease delays in care.

## Introduction

The global incidence of central nervous system (CNS) cancers is estimated to be 330,000 cases annually, and results in a death rate of 3.24 deaths per 100,000 person-years [1]. Even more significantly, this group of malignancies leads to 7.7 million disability adjusted life years (DALYs) lost annually worldwide [1]. Within Latin America, number of CNS cases and resultant mortalities and DALYs in Mexico fall second only to Brazil [1]. Despite the magnitude of this problem, worldwide clinical and research resources to improve oncologic care are biased towards high-income countries (HICs).

In the capital city of Mexico City, one of the main referral hospitals providing neuro-oncologic care is the Instituto Nacional de Neurología y Neurocirugía (INNN), where at least half of the presenting patients are referred from rural areas [2, 3]. Often patients with CNS and PNS tumors first present to rural facilities, where there is a lack of knowledge regarding neurosurgical pathologies, leading to a delay in diagnostic workup and referral to a tertiary center [4]. Additional delays often occur due to patients’ low educational level as well as financial and transportation limitations prevalent in many rural communities [5].

Although there have previously been several studies published looking at the overall epidemiology and clinical outcomes of surgical neuro-oncology patients treated at INNN, none have assessed the association between sociodemographic variables and advanced clinical presentation. Hence, we sought to not only describe the demographic and clinical epidemiology of surgical neuro-oncology patients treated at INNN in 2022, but to also identify sociodemographic factors association with more severe clinical presentation in this patient population. We hypothesized that lower education status, lower income, higher number of dependents and greater distance to INNN would be associated with more severe clinical presentations. Identifying these factors is key to determining which patient groups are most at risk and require planned targeted intervention to decrease delays in clinical presentation.

## Methods

### Study setting

The National Institute of Neurology and Neurosurgery of Mexico (INNN) is a tertiary public hospital in Mexico dedicated to clinical assistance, research and education in neurology, neurosurgery and psychiatry. The hospital was founded in 1964 in Mexico City, and currently admits adult patients > 14 years of age, coming primarily from the Mexico City area, which encompasses 1485 square kilometers (km) and 20.4 million urban inhabitants, with additional patients referred from peripheral Mexican States [6]. The hospital has a capacity of 126 hospital beds, 12 ICU beds, 4 operating theaters, 2 MRI, 1 CT scan and nuclear medicine [7]. The hospital has maintained an electronic medical records (EMR) system since 2018, which captures sociodemographic and clinical data for all patients admitted and treated.

### Data collection

The hospital EMR was reviewed to identify all neuro-oncology patients who underwent operation between January 1, 2022 and December 31, 2022. Information on patient sociodemographic variables, clinical history including symptoms, neurologic exam, imaging findings, management and outcome were abstracted from the EMR into an electronic password protected database by 5 trained research assistants, who were recently graduated Mexican medical students in their social service year. Since 1927 graduating Mexican medical students have been required to complete a year of primary care social service before obtaining their full medical license, which is intended to provide a safety net for underserved communities [8]. The outcomes of interest were duration of symptoms prior to presentation to INNN and tumor volume. Tumor volume was calculated the formula for the volume of an ellipsoid: 4/3π(A/2)(B/2)(C/2), where A, B, and C are the three diameters [9].

### Statistical analysis

Descriptive statistics were used to characterize the patient population and outcomes. There were no MRI images available for 23 patients and duration of symptoms was not provided for 2 patients. Thus those patients were include in the baseline descriptive analysis, but excluded from the univariate and multivariate analyses. Pearson’s correlation test to quantify the association between continuous sociodemographic variables (age, number of dependents, monthly income and distance traveled to INNN) and duration of symptoms prior to presentation to INNN and tumor volume. Wilcoxon rank-sum test was used to quantify association between categories variables with two groups (gender and primary provider status) and duration of symptoms prior to presentation to INNN and tumor volume. Kruskal–Wallis equality-of-populations rank test was used to quantify association between categories variables with more than two groups (education level and marital status) and duration of symptoms prior to presentation to INNN and tumor volume.

Univariate and multivariate logistic regression models were then used to further characterize association between sociodemographic variables and duration of symptoms prior to presentation to INNN (dichotomized into < = 37 weeks and > 37 weeks) and tumor volume (dichotomized into < = 35 mL and > 35mL), when controlling for confounders. The outcomes were dichotomized based on the median duration of symptoms and tumor volume in order to carry out logistic regression analyses. Stata 14.1 (StataCorp LLC, College Station, Texas, USA was used to carry out all analyses. Statistical significance was defined as a p-value < 0.05.

### Ethical approval

Ethical approval was obtained from Research Ethics Committee at the INNN (Protocol Number 22/23). There is no additional consent obtained from patients given all variables were collected retrospectively as part of routine clinical care at INNN, and no additional information was abstracted directly from patients.

## Results

### Patient demographics

There were a total of 366 neuro-oncology patients who underwent surgery between January 1, 2022 and December 31, 2022. Descriptive statistics for patients are found in Table 1. The median patient age was 48 (IQR: 17–83) and 60.1% were female (*n* = 220). The majority of patients (67.8%, *n* = 240) completed either completed primary (elementary), secondary (junior high school) or tertiary (high school) school. More than half of all patients were single (51.4%, *n* = 188) and 29.2% (*n* = 107) of patients endorsed being the primary provider for their family. The median number of dependents per patient was 4 (IQR: 2–50), while the median monthly income was 10269 Mexican pesos (MXN) (IQR: 2000–13500] and the median travel distance to INNN was 49 km (IQR: 22–174).

**Table 1:** Demographics of surgical neuro-oncology patients presenting to INNN, Jan 2022-Dec 2022 (*n* = 366)

	Total *n* (%)
**Age** (median [IQR])	48 [17–83]
**Gender**	
Male	146 (39.9%)
Female	220 (60.1%)
**Education Level**	
None	13 (3.5%)
Some Primary	36 (9.8%)
Completed Primary	85 (23.2%)
Completed Secondary	83 (22.7%)
Completed Tertiary	80 (21.9%)
Completed University	55 (15.0%)
Completed Vocational School	14 (3.6%)
**Marital Status**	
Single	188 (51.4%)
Married	126 (34.4%)
Divorced/Separated	24 (6.6%)
Widowed	28 (7.6%)
**Primary Provider**	
No	259 (70.8%)
Yes	107 (29.2%)
**# of Dependents** (median [IQR])	4 [2–5]
**Monthly Income**,** MXN** (median [IQR])	10,269 [2000–13500]
**Distance to INNN**, km (median [IQR])	49 [22–174]

### Patient clinical presentation & outcomes

Patient clinical presentation and outcomes are found in Table 2. The median length of symptom duration was 37 weeks (IQR: 12–110). The most common patient comorbidities were hypertension (25.6%, *n* = 94), diabetes mellitus (12.8%, *n* = 47) and being a current smoker (12.6%, *n* = 46). The most common presenting symptom was headache (60.9%, *n* = 223), followed by weakness (43.7%, *n* = 160) and vision changes (42.9%, *n* = 157). On neurologic exam, a total of 62.3% (*n* = 228) of patients presented with cranial nerve deficits, 43.4% (*n* = 159) presented with motor weakness and 25.1% (*n* = 92) with sensory changes. Computed tomography (CT) and magnetic resonance imaging (MRI) were obtained in the majority of patients, with 91% (*n* = 333) and 94.8% (*n* = 346) obtaining these imaging modalities, respectively. The median tumor volume was 35mL (IQR: 13–82).

**Table 2:** Clinical presentation and outcomes of surgical neuro-oncology patients presenting to INNN, Jan 2022-Dec 2022 (*n* = 366)

	Total *n* (%)
**Length of Symptom Duration**,** wk** (median [IQR])	37 [12–110]
**Medical Comorbidities**	
Hypertension	94 (25.6%)
Diabetes	47 (12.8%)
Current Smoker	46 (12.6%)
Hyperlipidemia	28 (7.6%)
Coronary Artery Disease	7 (1.9%)
Chronic Obstructive Pulmonary Disease	4 (1.1%)
HIV +	3 (0.8%)
**Presenting Symptoms**	
Headache	223 (60.9%)
Weakness	160 (43.7%)
Vision Changes	157 (42.9%)
Sensory Changes	88 (24.0%)
Confusion	85 (23.2%)
Seizures	77 (21.0%)
Dysarthria	49 (13.4%)
**Presenting Signs**	
Cranial Nerve Deficits	228 (62.3%)
Motor Weakness	159 (43.4%)
Sensory Changes	92 (25.1%)
**Imaging Study Obtained**	
CT	333 (91.0%)
CTA	15 (4.1%)
MRI	346 (94.8%)
MRA	4 (1.1%)
**Tumor Volume**,** mL** (median [IQR])	35 [13–82]
**Outcome**	
Discharged	335 (91.6%)
Transferred	2 (0.6%)
Died	29 (7.9%)
**Rehabilitation Needs**	
Physical	133 (36.3%)
Occupational	109 (29.8%)
Speech	29 (8.0%)
None	95 (24.9%)

**Fig. 1:**
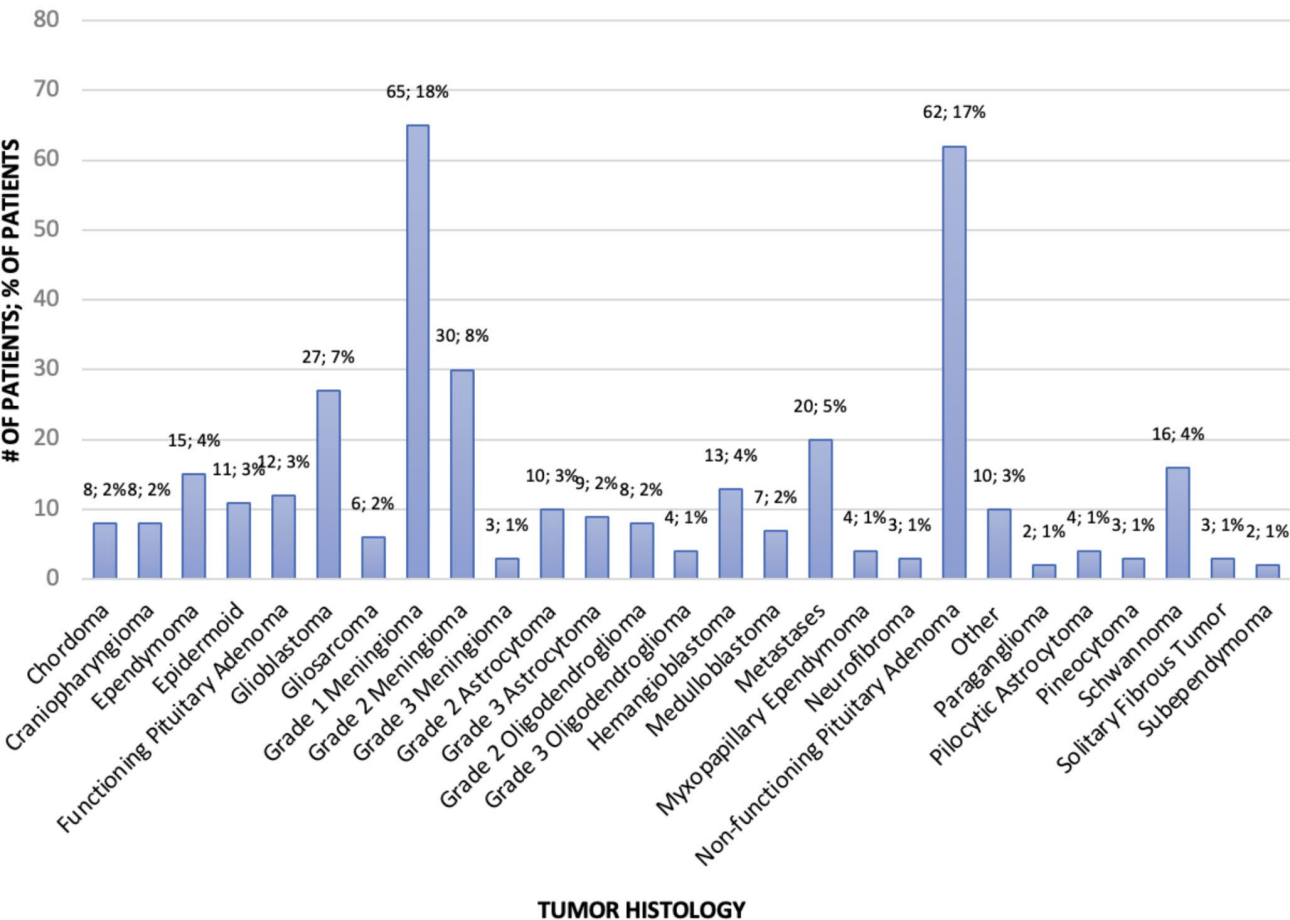
Histologic breakdown of tumor types operated on at INNN, Jan 2022 - Dec 2022 (*n* = 366)

The most common tumor treated was a grade 1 meningioma (18%, *n* = 65), followed by non-functioning pituitary adenoma (17%, *n* = 62), and grade 2 meningioma (8%, *n* = 30) (Fig. 1). A total of 22.7% (*n* = 83) of patients had glial tumors, which included pilocytic astrocytoma, grade 2 and 3 astrocytoma, grade 2 and 3 oligodendroglioma, glioblastoma and gliosarcoma, ependymoma. Of all glial tumors, 24% (*n* = 20) and 22.9% (*n* = 19) carried the IDH1 or IDH2 mutation, respectively. Other molecular classifications, including ATRX, TERT, 1p19q, and MGMT methylation status were not readily available. In-hospital mortality rate was 7.9% (*n* = 29). The majority of patients (74.1%, *n* = 271) required some form of rehabilitation after surgery.

### Association between sociodemographic variables and advanced presentation

There was a statistically significant association between being a primary provider and shorter duration of symptoms at time of presentation (Table 3). When looking at the relationship between sociodemographic variables and tumor volume, there was a weak but statistically significant correlation between age and tumor volume, with younger patients more likely to present with larger tumor volumes (*r*=-0.11, *p* = 0.04). Additionally, patients who were divorced/separated were more likely to present with larger tumor volumes, compared to patients with other marital statuses (*p* = 0.01). Finally, being a primary provider was associated with small tumor volumes (*p* = 0.03) (Table 4).

**Table 3:** Association between sociodemographic variables and duration of symptoms among neuro-oncology patients presenting to INNN, Jan 2022-Dec 2022 (*n* = 366)

	Duration of Symptoms, wk median [IQR]	*P*-value
Age*	0.059	0.27
**Gender**		
Male	32 [12–104]	0.54
Female	46 [9-148]	
**Education Level**		
None	28 [2–90]	0.31
Some Primary	22 [10.5–72]	
Completed Primary	32 [12–104]	
Completed Secondary	66 [18–156]	
Completed Tertiary	52 [8-116]	
Completed University	24 [8-148]	
Completed Vocational School	29.5 [15–156]	
**Marital Status**		
Single	35.5 [12–116]	0.93
Married	52 [12–156]	
Divorced/Separated	48 [8-93.5]	
Widowed	24 [8-90.5]	
**Primary Provider**		
No	52 [13–156]	**0.04**
Yes	24 [8–92]	
# of Dependents*	0.064	0.23
**Monthly Income**, MXN*	0.0006	0.99
**Distance to INNN**, km*	-0.031	0.55

*Pearson’s correlation test

**Table 4:** Association between sociodemographic variables and tumor volume among neuro-oncology patients presenting to INNN, Jan 2022-Dec 2022 (*n* = 366)

	Tumor Volume, mL median [IQR]	*P*-value
Age*	-0.109	**0.04**
**Gender**		
Male	40.7 [15.4–92.0]	0.14
Female	34.5 [11.4–79.3]	
**Education Level**		
None	59.0 [23.7–82.5]	0.53
Some Primary	40.3 [14.7–88.2]	
Completed Primary	35.1 [14.4–74.3]	
Completed Secondary	31.8 [9.4–92.8]	
Completed Tertiary	40.9 [18.2–77.1]	
Completed University	22.9 [10.5–64.2]	
Completed Vocational School	16.0 [11.5–68.3]	
**Marital Status**		
Single	40.3 [13.0-82.4]	**0.02**
Married	25.9 [12.5–68.4]	
Divorced/Separated	68.3 [45.6-111.2]	
Widowed	35.3 [12.9–76.1]	
**Primary Provider**		
No	40.7 [14.9–85.9]	**0.03**
Yes	25.1 [9.4–71.3]	
# of Dependents*	-0.009	0.86
**Monthly Income**, MXN*	-0.079	0.15
**Distance to INNN**, km*	0.028	0.60

*Pearson’s correlation test

In univariate analyses looking at the odds of presenting with symptom duration for > 37 weeks, having a higher number of dependents was associated with increased odds of presenting with longer symptom duration (OR: 1.2, 95%CI: 1.06–1.35, *p* = 0.005). This association remained statistically significant on multivariate analyses, when controlling for patient age, gender, education level, marital status, primary provider status, monthly income and distance traveled to INNN (aOR: 1.19, 95%CI: 1.03–1.36, *p* = 0.01) (Table 5).

**Table 5:** Logistic regression model of sociodemographic variables associated with duration of symptoms > 37 weeks among neuro-oncology patients presenting to INNN, Jan 2022-Dec 2022 (*n* = 366)

	OR (95% CI)	*P*-value	Adjusted OR (95% CI)	*P*-value
**Age**	0.99 (0.98–1.01)	0.32	1.00 (0.99–1.02)	0.73
**Gender**				
Male	Baseline	-	Baseline	-
Female	1.05 (0.69–1.59)	0.83	0.99 (0.62–1.60)	0.98
**Education Level**				
None	Baseline	-	Baseline	-
Some Primary	0.74 (0.21–2.67)	0.65	0.80 (0.21–2.95)	0.73
Completed Primary	0.99 (0.31–3.19)	0.99	1.08 (0.32–3.65)	0.90
Completed Secondary	1.82 (0.56–5.91)	0.32	1.94 (0.56–6.72)	0.30
Completed Tertiary	1.26 (0.39–4.08)	0.70	1.31 (0.37–4.59)	0.67
Completed University	1.05 (0.31–3.52)	0.94	1.37 (0.37–5.06)	0.64
Completed Vocational School	0.88 (0.19-4.00)	0.86	0.95 (0.19–4.55)	0.95
**Marital Status**				
Single	Baseline	-	Baseline	-
Married	1.12 (0.71–1.77)	0.61	1.01 (0.61–1.69)	0.96
Divorced/Separated	1.21 (0.51–2.83)	0.67	1.37 (0.56–3.37)	0.48
Widowed	0.66 (0.29–1.49)	0.32	0.72 (0.28–1.83)	0.49
**Primary Provider**				
No	Baseline	-	Baseline	-
Yes	0.65 (0.41–1.03)	0.07	0.65 (0.39–1.10)	0.11
**# of Dependents**	1.20 (1.06–1.35)	**0.005**	1.19 (1.03–1.36)	**0.01**
**Monthly Income**,** MXN**	1.00 (0.99-1.00)	0.35	0.99 (0.99-1.00)	0.87
**Distance to INNN**,** km**	1.00 (0.99-1.00)	0.47	1.00 (0.99-1.00)	0.55

In the univariate analyses looking at the odds of presenting with a tumor > 35mL in volume, being divorced/separated was associated with increased odds of presenting with a tumor volume > 35mL (OR: 2.96, 95%CI: 1.04–8.41, *p* = 0.04), while being a primary provider (OR: 0.58, 95%CI: 0.36–0.94, *p* = 0.03) and having a higher average monthly income (OR: 0.99, 95%CI: 0.99–0.99, *p* = 0.03) was associated with decreased odds of presenting with a tumor volume > 35mL. These associations remained statistically significant on multivariate analyses with divorced/separated status (OR: 3.11, 95%CI: 1.04–9.31, *p* = 0.04), being associated with increased odds of presenting with tumors > 35mL in volume and being a primary provider (OR: 0.45, 95%CI: 0.26–0.79, *p* = 0.01) and having a higher average monthly income (OR: 0.99, 95%CI: 0.99–0.99, *p* = 0.03) being associated with decreased odds of presenting with tumors > 35mL in volume, when adjusting for age, gender, education level, number of dependents and distance traveled to INNN (Table 6).

**Table 6:** Logistic regression model of sociodemographic variables associated with tumor volume > 35 mL among neuro-oncology patients presenting to INNN, Jan 2022-Dec 2022 (*n* = 366)

	OR (95% CI)	*P*-value	Adjusted OR (95% CI)	*P*-value
**Age**	0.99 (0.98–1.01)	0.64	1.00 (0.99–1.02)	0.74
**Gender**				
Male	Baseline	-	Baseline	-
Female	0.89 (0.58–1.38)	0.61	0.67 (0.40–1.10)	0.11
**Education Level**				
None	Baseline	-	Baseline	-
Some Primary	1.07 (0.28–4.06)	0.92	1.13 (0.29–4.39)	0.86
Completed Primary	0.71 (0.21–2.44)	0.59	0.68 (0.19–2.38)	0.54
Completed Secondary	0.65 (0.19–2.21)	0.49	0.64 (0.18–2.29)	0.49
Completed Tertiary	0.96 (0.28–3.30)	0.95	1.11 (0.30–4.09)	0.88
Completed University	0.46 (0.13–1.67)	0.24	0.61 (0.16–2.39)	0.48
Completed Vocational School	0.32 (0.06–1.64)	0.17	0.36 (0.07–1.92)	0.23
**Marital Status**				
Single	Baseline	-	Baseline	-
Married	0.68 (0.42–1.09)	0.11	0.64 (0.38–1.08)	0.10
Divorced/Separated	2.96 (1.04–8.41)	**0.04**	3.11 (1.04–9.31)	**0.04**
Widowed	0.99 (0.44–2.24)	0.99	0.85 (0.33–2.18)	0.73
**Primary Provider**				
No	Baseline	-	Baseline	-
Yes	0.58 (0.36–0.94)	**0.03**	0.45 (0.26–0.79)	**0.01**
**# of Dependents**	1.00 (0.91–1.10)	0.96	1.08 (0.95–1.24)	0.25
**Monthly Income**,** MXN**	0.99 (0.99–0.99)	**0.03**	0.99 (0.99–0.99)	**0.03**
**Distance to INNN**,** km**	1.00 (0.99-1.00)	0.24	1.00 (0.99-1.00)	0.14

## Discussion

In this retrospective study, we described the demographic and clinical epidemiology of neuro-oncology patients treated at a public tertiary referral hospital in Mexico City and identified sociodemographic factors associated with more severe clinical presentation in this patient population. We found that for the 366 patients who underwent surgery at INNN in 2022, sociodemographic variables such as marital status, being a primary provider, number of household dependents and income level were associated with duration of symptoms and tumor volume at presentation. This was the first study to identify possible factors associated with advanced clinical presentation among neuro-oncology patients treated at the main tertiary referral public hospital in Mexico City.

Statistically robust data regarding the incidence of cancer in Mexico is largely unknown due to a lack of a national cancer registry [2]. Prior studies have attempted to describe the neuro-oncology patient population in Mexico and at INNN specifically, given the institute is the main public referral hospital in the country. In a study conducted at INNN over a 7 years period, the mean age of brain tumor patient was 51 years of age, with the majority being male [10]. The most common male occupation was agriculture while the most common female occupation was a homemaker, and more than half of all patients lived in rural areas [10]. Our patient population was of similar age; however, the majority of patients operated on in 2022 were female. We did not collect information of specific occupation, but almost one third of our patients were primary providers for their family and patients lived on average more than 150 km away from INNN. The most frequent presenting symptoms were similar to those of patients admitted to tertiary hospitals in Sub-Saharan Africa – headache, vision disturbances and weakness [11].

We found that the distribution of different CNS and PNS cancer types was similar compared to previously published descriptions [12]. Hernandez-Hernandez et al. reviewed all patients treated for CNS tumors at INNN over the past 52 years and reported that the most common pathologies were meningiomas (20%), pituitary adenomas (18%), glial tumors (17%), with metastases only making up 4% of tumor cases [13]. Meanwhile, a different study at INNN conducted over a 7 year period found that the majority of patients presenting to INNN had either a pituitary adenoma or glial tumors [10]. Within our patient cohort, we were able to collect more detailed information on tumor grades and various molecular markers than what has been previously published in Mexico, with the most common tumor treated being grade I meningioma, followed by non-functioning pituitary, and grade II meningioma. Glioblastoma patients made up 7% of the population; however, more than one fifth of all patients presented with some type of glial tumor. We were able to reliably check IDH1/2 mutant status on our patient cohort, although IDH mutation status is not commonly performed outside of Mexico City, and determination of MGMT promoter methylation is not currently feasible [2].

Due to a multitude of reasons, delays in diagnosis and treatment of neuro-oncologic patient are common worldwide but are particularly pronounced in underserved countries. In one study from Nigeria, authors found that the median duration of patient symptoms prior to presentation to a healthcare facility was 2 years, and ranged from 2 months to 5 years [11]. Our median pre-presentation symptom interval was slightly less at 37 weeks, with a range of 0 days to 31 years. While others have found that female gender was associated with longer symptom duration, we found that a having a higher number of household dependents was associated with longer duration of symptoms at time of presentation [11].

Since patient recall of their clinical history is subjective, variables such as neurologic exam at admission and tumor volume can be considered as proxies for presentation delay. Interestingly, patients in our cohort who were primary providers for their families were more likely to present with smaller tumor volumes. Although no previous studies have looked at association between being the primary provider for the family and severity of clinical presentation, Yoshida et al. found that primary providers (‘breadwinners’) had superior clinical outcomes and were 15 times more likely to return to work within one year after awake glioma surgery [14, 15]. Within out cohort, marital status also appeared to play a role in patient presentation with divorced/separated patients being more likely to present with larger tumors, compared to single and married patients. This is in contrast to findings from Sutton et al., who found no association between marital status an acuity of clinical presentation in a single institution study of rectal cancer patients [16]. Finally, patients who had a higher monthly income presented with smaller tumor volumes, mirroring findings from an observational study of patients receiving stereotactic radiation for brain metastases at Wake Forest Comprehensive Cancer Center between 2000 and 2013, which demonstrated that lower median income was associated with more severe neurologic symptoms at presentation [17]. Additionally, within our cohort, patients with a higher number of household dependents were more likely to present with a longer duration of symptoms, perhaps pointing to other life stressors that may have prevented them from seeking care earlier.

Overall, our study found that multiple sociodemographic variables are associated with more severe clinical presentation among neuro-oncology patients treated at INNN. Lesser number of dependents, marriage, being a primary provider, and higher monthly income were all shown to be protective factors associated with less severe clinical presentation, highlighting the importance of social determinants of health and health care seeking behavior. Increasing awareness of common presentations of neurologic conditions at the laymen level, via public health campaigns, may decrease the time to healthcare facility presentation. Additionally, offloading the economic burden of presenting for a clinic evaluation, by providing transportation vouchers, may be a possible solution [18]. However, it is important to note that even in cases where access to the healthcare system is finally obtained, it may take months to obtain evaluation from a specialist due to limited medical specialists [5]. In one study, presentation to a neurological specialist was delayed in 21% of cases because general practitioners did not consider intracranial tumor/cranial scan despite persistent symptoms [11]. One solution may be for neurosurgeons to offer medical education symposia on common neurologic conditions and recommended workup via telehealth conference platforms to community healthcare providers. Further prospective studies involving detailed patient and provider interviews assessing their personal reasons for and experiences with presentation, diagnostic and treatment delays are necessary to decipher systemic causes for this issue prior to planning targeted interventions.

Our study has several limitations. All clinical patient information was obtained retrospectively from medical records, hence certain clinical variables, such as duration of clinical symptoms, were subject to recall bias. Only information available in the patient record was included in our analyses, and thus certain confounders may have been missed. Additionally, since this was a hospital-based study set in a major urban center, it has limitations in generalizability to more rural populations throughout Mexico. Moreover, given this was a database-based study, long-term follow up post hospital discharge was limited. Finally, this study only included those neuro-oncology patients who underwent surgery, thus findings do not apply to patients who were not surgical candidates.

## Conclusion

This is the first study to identify sociodemographic factors associated with more severe clinical presentation in this patient population. Multiple sociodemographic variables are associated with more severe clinical presentation among this patient population, and further studies are needed in order to decern specific causes for healthcare delays in this patient cohort. Identifying sociodemographic factors associated with advanced clinical presentation will enable providers to target future interventions towards the most vulnerable patient population in hopes of improving their clinical outcomes.

## Data Availability

Data may be obtained by individual request and is not publicly available.
